# Albumin and multiple sclerosis

**DOI:** 10.1186/s12883-016-0564-9

**Published:** 2016-04-12

**Authors:** Steven M. LeVine

**Affiliations:** Department of Molecular and Integrative Physiology, University of Kansas Medical Center, Kansas City, KS USA

**Keywords:** Albumin, Albumin quotient, Blood–brain barrier, Cerebrospinal fluid, Experimental autoimmune encephalomyelitis, Macrophages, Multiple sclerosis, Reactive nitrogen species, Reactive oxygen species

## Abstract

Leakage of the blood–brain barrier (BBB) is a common pathological feature in multiple sclerosis (MS). Following a breach of the BBB, albumin, the most abundant protein in plasma, gains access to CNS tissue where it is exposed to an inflammatory milieu and tissue damage, e.g., demyelination. Once in the CNS, albumin can participate in protective mechanisms. For example, due to its high concentration and molecular properties, albumin becomes a target for oxidation and nitration reactions. Furthermore, albumin binds metals and heme thereby limiting their ability to produce reactive oxygen and reactive nitrogen species. Albumin also has the potential to worsen disease. Similar to pathogenic processes that occur during epilepsy, extravasated albumin could induce the expression of proinflammatory cytokines and affect the ability of astrocytes to maintain potassium homeostasis thereby possibly making neurons more vulnerable to glutamate exicitotoxicity, which is thought to be a pathogenic mechanism in MS. The albumin quotient, albumin in cerebrospinal fluid (CSF)/albumin in serum, is used as a measure of blood-CSF barrier dysfunction in MS, but it may be inaccurate since albumin levels in the CSF can be influenced by multiple factors including: 1) albumin becomes proteolytically cleaved during disease, 2) extravasated albumin is taken up by macrophages, microglia, and astrocytes, and 3) the location of BBB damage affects the entry of extravasated albumin into ventricular CSF. A discussion of the roles that albumin performs during MS is put forth.

## Background

Multiple sclerosis (MS) is believed to result from an underlying autoimmune mechanism that leads to the development of central nervous system (CNS) lesions that eventually cause sensory and motor symptoms [1, 2]. The majority of patients experience a relapsing remitting type of MS (RRMS), but over time the condition frequently transitions into a progressive form of disease [1, 2]. Active demyelinating lesions can result from pro-inflammatory immune cells migrating across the vasculature into the CNS, and this process is affiliated with a breakdown of the blood–brain barrier (BBB) [3–5]. Besides this association with immune cells, BBB leakage can result in the extravasation of plasma components that cross damaged vessels and enter the CNS. Perivascular immune cells and vascular leakage occur in both acute and chronic MS lesions [6], and damage to the BBB may be relatively persistent since vascular changes can be present without concurrent inflammatory cells, e.g., after their departure, and plasma proteins can be present in older, inactive lesions [7, 8]. Vascular leakage can also occur in normal appearing white matter [9–12], and although BBB disruption usually occurs in the development of a new lesion, evidence suggests that it might also arise following neurodegeneration in MS [11, 13].

Serum albumin represents ~50 % of the proteins in plasma where it has a half-life of ~15–20 days [14, 15]. Serum albumin is a ~66.4 kDa, heart shaped protein that has a variety of functions including being the primary plasma component affecting oncotic pressure, transporting fatty acids, carrying some hormones, influencing drug pharmacokinetics, binding metals and heme, and acting as an anti-oxidant [14, 15]. Given its high concentration in the plasma, albumin would be expected to access CNS tissue following the breakdown of the BBB that occurs during MS. Once in the CNS, evidence suggests that albumin is not an inert bystander, but rather is positioned to impact the disease course given its relative abundance, its molecular properties, and the reoccurring and/or chronic nature of BBB disruption during disease. In this review, a discussion is put forth about various protective and pathogenic mechanisms of albumin relative to MS.

## Review

### A compromised BBB is a common occurrence in MS

MRI detection of gadolinium (Gd) enhancing lesions is one measure used for the diagnosis of MS and for monitoring disease activity [16, 17]. Gd does not enter the brain when there is an intact BBB, but when there is a breach in the BBB, it appears as a local enhancement. Gd-enhancing lesions occur most commonly in RRMS and secondary progressive MS (SPMS), but can also occur in benign MS, clinically isolated syndrome, pediatric MS, and primary progressive MS (PPMS) (more commonly, enhancing lesions occur earlier in the course of PPMS), and enhancements are usually considered a marker of active lesions [18–23]. Lesions can have different appearances, with nodular or uniform Gd enhancements representing new lesions associated with BBB leakage while ring or arc enhancements suggest older lesions [24, 25]. Based on numerous Gd studies, the breakdown of the BBB is relatively common occurrence during MS, and a triple dose of Gd may increase the ability to detect lesions [18, 19, 26] with one study revealing an average of 5.17 (median 3.38) enhancing lesions per month per patient (37 RRMS, 3 SPMS) with an average of 3.37 (median 2.5) of the lesions being new enhancements per month per patient when using a delay of 20 min for imaging after the administration of a triple dose of Gd [27].

### BBB damage in MS results in excess albumin gaining access to the CNS

BBB leakage results in the extravasation of leukocytes, some red blood cells (RBCs), and plasma proteins into the CNS in both experimental autoimmune encephalomyelitis (EAE) [28–31], an animal model of MS, and MS tissue [32–35]. Since albumin is the predominant protein in plasma, it would be expected to be among the proteins that gain access to the CNS. For instance, proteomic [30, 36], immunohistochemical [37–40], immunoblotting [41], Evan’s blue labeled albumin [42], CSF [43, 44], and radiolabeled albumin [45] studies in EAE subjects revealed that albumin enters the CNS during disease. Albumin extravasation precedes both cellular inflammation and clinical signs, and it occurs initially around vessels, i.e., perivascular space in subpia, and then spreads diffusely into the CNS [38].

In human MS tissue, albumin was found widely dispersed in the CNS in immunohistochemical studies of mostly chronic MS cases [46] and inactive plaques [7], and it was revealed by proteomics studies in some chronic plaques/lesions [35, 47]. It is somewhat surprising that albumin has not been detected or described more frequently in active MS plaques, especially since a low dose of gadofosveset trisodium, a Gd compound that binds albumin reversibly, revealed more enhancements, albeit at a 4 h time point, compared to gadoterate meglumine (only < 4 % is bound to plasma proteins in vitro [48]), at a more standard 4 min time point [49]. Some possible reasons why albumin has not been detected or described more frequently in active plaques are as follows. In contrast to fibrinogen, which is more discretely localized around leaky vessels and readily detected in active plaques [46, 50, 51], the distribution of albumin appears to be more widespread following BBB leakage [7, 40, 46] which could make it more difficult to detect by immunohistochemistry. Additionally, albumin appears to be cleared relatively quickly from the CNS following extravasation [40]. Albumin that was labeled with gold, for detection via electron microscopy, has been shown to be rapidly taken up by subarachnoidal macrophages [52], which is relevant since macrophages are a substantial component of active lesions, but less so in inactive plaques [53–55], which interestingly have more detectable albumin [7]. Albumin is also proteolytically cleaved during acute phases of RRMS [56], which could make it more difficult to detect by immunohistochemistry.

CSF albumin levels or the albumin quotient (albumin in CSF/albumin in serum) were elevated in ~12–23 % of MS cases [57–62]. An elevated level of albumin in the CSF, or an elevated albumin quotient, is thought to be a measure of blood-CSF dysfunction in MS [63], and it has been used as an indicator of BBB permeability [58, 60, 62]. The albumin quotient is less sensitive than Gd MRI for detecting BBB disruption especially in supraspinal lesions [64], and the albumin quotient is sensitive to the subject’s age [65].

Detection of an elevation of albumin in CSF would be expected to be dependent on the timing and location of BBB leakage. Albumin rapidly diffuses through the rat brain with a disappearance half-life of ~12 h [66]. After infusion into the caudate nucleus of the rat, only a relatively small percentage of the infused albumin ended up in the CSF at the cisterna magna [66] indicating that albumin can exit the CNS by a route(s) other than through the ventricular system [64, 66]. Indeed, following an intracerebral injection of ovalbumin in mice, some digestion products found their way to the cervical lymph node [67]. Although caution about extrapolation of these results is warranted, since ovalbumin is antigenically different than mouse albumin, ovalbumin products were observed in the lymph node at 2, 4, and 8 h, and 7 days, following an intracerebral injection [67]. A small percentage of the digestion products remained in the CNS, i.e., in CD11B/MAC-1^+^ cells (monocytes), over long periods, e.g., 4 weeks after injection, but these were largely present around the injection site [67]. In addition to different exit routes, it is likely that a change in CSF albumin levels during MS is sensitive to the location of the BBB leakage with spinal lesions and possibly circumventricular lesions potentially giving rise to the greatest elevation [64]. It is also relevant to note that CSF albumin levels are dependent on the rate of albumin influx from multiple sources (e.g., transport from blood to CSF in the choroid plexus, BBB leakage, and possibly synthesis within the CNS) as well as the rate of efflux (e.g., turnover or flow of CSF). In normal individuals, the major source of CSF albumin is its transport from the blood, via binding glycoprotein receptors on epithelial cells in the choroid plexus, and subsequent transfer into the ventricular CSF [68]. In MS, changes in albumin transfer through the choroid plexus, BBB leakage, and an increased CNS synthesis of albumin could all affect the CSF albumin concentration.

The volume of CSF is renewed rapidly in the 3 month (11 times/day) and 19 month (10.8 times/day) rat, and more slowly in the 30 month rat (3 times/day) [69]. In the human, CSF is renewed ~4 times/day [69]. The clearance of albumin in the CSF is relatively fast, e.g., in the mouse, albumin administered to the CSF resulted in its rapid clearance, e.g., only ~6 % remained in the CSF at 1 h [70]. During disease, the rate of albumin turnover/clearance could be altered. The rate of CSF flow through the aqueduct of Sylvius is decreased in MS patients [71], and a decreased flow could alter albumin concentrations [72], e.g., if the rate of albumin influx was constant but flow decreased, then the albumin concentration could increase.

Additionally, albumin catabolism could affect its concentration in CSF. Albumin is rapidly sequestered by subarachnoidal macrophages [52] and it can be taken up by astrocytes, microglia and neurons [73–76]. Furthermore, albumin fragments were found in the CSF of RRMS subjects during an acute phase, and these were differentially observed compared to CSF from control and Leber hereditary optic neuropathy subjects [56]. It has been suggested that albumin fragmentation is due to protease action by infiltrating immune cells [56]. Cytotoxic lymphocytes and macrophages produce proteases [77, 78], that in theory could act on albumin [56], but albumin fragmentation has been observed in the CSF from hydrocephalus [79], which results in a different inflammatory profile than MS. Data from a study on bronchoalveolar lavage fluid suggested that matrix metalloproteinase 3 (MMP-3) was responsible for digesting albumin, although other proteases may have also been involved in the fragmentation [80]. MMP-3 plasma levels are elevated in MS compared to control subjects [81] and MMP-3 levels in serum are increased during a relapse compared to remission [82]. MMP-3 is produced by a variety of CNS cells, e.g., microglia/macrophages, pericytes, astrocytes, endothelial cells, and ischemic neurons [83–85], and in MS MMP-3 expression has been observed in microglia/macrophages, astrocytes, and microvessels [86]. It is likely that multiple proteases act on albumin during MS, but independent of the cause of albumin fragmentation, the detection of albumin in CSF (or serum) by electrophoresis could miss these fragments resulting in an underrepresentation of the amount of albumin in CSF from MS subjects. Thus, albumin levels might be elevated in a greater percentage of MS patients if fragmented albumin was detected by the assay.

Given that multiple factors can influence CSF albumin levels, an assay measuring CSF albumin levels would be expected to be an imprecise way to assess BBB leakage, or blood-CSF dysfunction, in MS. A summary of influencing factors include: BBB damage can occur anywhere in the CNS and the location of BBB leakage can affect the entry of albumin into the ventricular system; the rate of albumin or CSF transport or production may be altered during disease; albumin leaked into the CNS can exit by means other than into the ventricular system; the timing of CSF collection may not exactly coincide with the maximal peak of BBB leakage; extravasated albumin can be catabolized by immune or CNS cells; and the assay likely misses albumin that has been digested. Thus, the determination that ~12–23 % of MS cases have an elevated CSF albumin or albumin quotient, and therefore a leaky BBB [57–62], could be inaccurate. If the albumin quotient is flawed, then this has the potential to impact the CSF IgG index, which is a commonly used as a measure of IgG production within the CNS, since the index represents the ratio of CSF IgG to CSF albumin divided by the ratio of serum IgG to serum albumin. However, at present there is little data that directly addresses whether the albumin quotient or IgG index are influenced by the factors listed above, and if they are influenced, it is possible that the impact would not be sufficient to affect these measures in a substantial manner. Thus, additional studies are needed to resolve this potential issue.

In RRMS, there was a trend between decreased CSF flow and relapse rate in the preceding year [71], and an elevated albumin quotient at the time of a first clinical event, thought to be related to MS, is associated with a greater reduction in volume of several brain structures within 2 years of the clinical event [62]. This raises the possibility that the inflammation accounting for this elevation in CSF albumin (or reduced CSF flow), or the leaked albumin or other plasma component entering the brain, caused more severe pathological changes.

### Protective roles of albumin during MS

Numerous studies have established that reactive oxygen species (ROS) and reactive nitrogen species (RNS) are participants in EAE and MS pathogenesis. Elevation of markers of ROS and RNS presence have been observed in leukocytes, serum, CSF, and CNS tissue of EAE and MS subjects [40, 87–99]. Given the high concentration of albumin in plasma, and the leakage of the BBB that occurs during disease, albumin would be expected to be an abundant substrate for ROS and RNS in MS. Thus, albumin could have a protective effect on the disease course by acting as a target for reactive molecules that otherwise would have greater access to damage more important biomolecules.

**Fig. 1:**
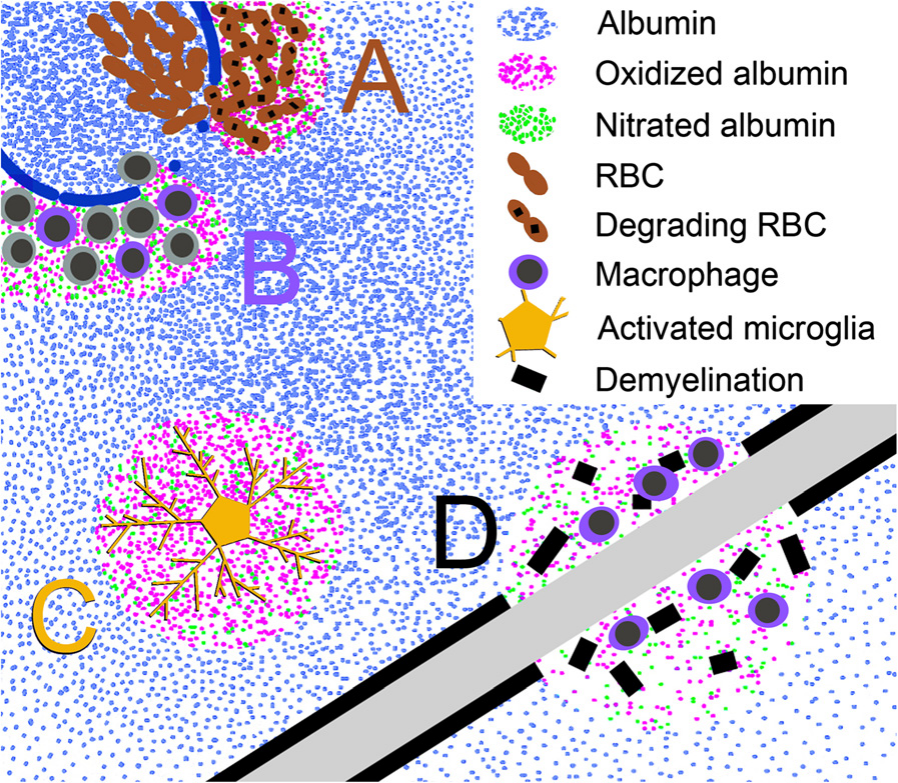
Protective mechanisms by albumin in the CNS during active MS disease. **a** As a consequence of BBB damage, albumin (*light blue dots*) becomes extravasated and micro-hemorrhages (RBCs, *rust color*) can occur around vessels (*royal blue*) in MS CNS tissue. The RBCs breakdown (*rust color with black spots*) and heme/iron is released which can catalyze oxidation and nitration reactions. Albumin can bind heme and iron, which limits their ability to promote tissue damage. In addition, albumin is often the recipient of toxic species that are generated (*pink dots* – oxidized albumin; *green dots* – nitrated albumin), thereby protecting other CNS biomolecules. Albumin bound to heme may also detoxify ROS and RNS. **b** Inflammatory cells cross the BBB, and can be a source of ROS and RNS, particularly macrophages (e.g., M1 macrophages) (*purple cells*). The colocalization of albumin with macrophages positions albumin to be a target of ROS and RNS. **c** Besides macrophages, microglia that become activated during MS (*yellow cell*) can be a source of ROS and RNS. Extravasated albumin becomes a target for these toxic species and thereby limiting tissue damage to other important molecules in the CNS. **d** Myelin is a site of iron concentration, and during demyelination (*black line fragments*) iron is released. This iron can catalyze oxidation and nitration reactions together with inflammatory cells, e.g., macrophages. Albumin can be a recipient of reactive molecules and becomes modified. Note, the concentration of albumin would become diluted (*top left to lower right*) in relation to the distance from the site of the damaged (leaky) BBB, at least until an equilibrium is reached

Serum albumin has anti-oxidant properties, in particular, Cys34 (which is conserved among mammals) scavenges free radicals, and six methionine residues can become oxidized [15, 100]. Besides oxidation, human serum albumin is a recipient of nitration and nitrosylation reactions [15, 100–102], and S-nitrosylated human serum albumin can serve as a transport mechanism for nitric oxide [103, 104]. Since nitric oxide, i.e., generated from iNOS, may have a pathogenic role in MS [105], it is possible that the transportation of nitric oxide by albumin could be beneficial or deleterious depending on whether it was being removed or delivered, respectively. Albumin also binds metals and heme. Included among the metals that bind albumin are copper [106] and iron [107]. These metals, and heme, can catalyze the formation of hydroxyl radical, but less so when bound to albumin, and the hydroxyl radical catalyzed from a metal bound to albumin is thought to largely interact with albumin itself rather than damaging other biologically relevant molecules [14, 15] (Fig. 1). Iron and heme can also catalyze the nitration of proteins [108–110], but when heme is bound to human serum albumin [103, 111–114] it may facilitate the detoxification of ROS and RNS [115–118]. The Cys34 on albumin can also form disulfide interactions with glutathione, cysteine, or homocysteine, while Arg410 and Lys525 are main targets of glycation [15, 119] (discussed below).

Iron and hemoglobin (e.g., extravasated RBCs) have been detected around damaged vessels in EAE and MS tissue [28, 32, 33, 40, 98, 120–122] (Fig. 1). This is also where extravasation of albumin originates thereby resulting in a high concentration at this site in comparison to albumin diffusing away from leaky vessels to other CNS structures and becoming diluted in the process (Fig. 1). Since iron and heme can catalyze reactions leading to oxidation and nitration [108–110, 123–125], it indicates that albumin is positioned to be an early recipient of these reactive species during BBB leakage (Fig. 1). Interestingly, nitrated proteins have been detected around vessels in EAE and MS [40, 126–128], and it has been put forth that extravasated albumin from leaky vessels is a main target for nitration during disease [40]. In addition, extravasated albumin is positioned to directly bind iron and heme originating from extravasated RBCs or liberated as a consequence of ongoing tissue damage, e.g., demyelination since iron can be abundant within myelin [129] (Fig. 1). This interaction with albumin would serve to limit the ability of iron and heme to form toxic radicals [14, 15], and act to possibly detoxify ROS and RNS [115–117]. The intravenous administration of albumin to rats with subarachnoid hemorrhage, modeled via endovascular perforation, resulted in improved behavioral outcomes and limited BBB leakage, and one mechanism for this effect could be the binding of heme and/or iron to albumin thereby limiting ROS and RNS damage [130].

Inflammatory cells, i.e., macrophages and reactive microglia, can also produce RNS and ROS during EAE and MS [88, 131–135]. Given that macrophages are a main participant in active lesions [53–55, 136], extravasated albumin could be a partial buffer limiting the spread of damage induced by these RNS and ROS to the surrounding tissue (Fig. 1). Analogously, myeloperoxidase has an elevated expression in macrophages and microglia in MS and it is thought to promote tissue damage [133, 137–139]. Since myeloperoxidase causes oxidation, nitration and nitrosylation to human serum albumin [140], it raises the possibility that albumin absorbs some of the toxic products of myeloperoxidase and thereby protecting more important biomolecules (Fig. 1).

In CSF from MS subjects, albumin becomes modified [141] and albumin fragments become carbonylated (which is thought to result from oxidative stress) [142]. Furthermore, ischemia modified albumin (IMA), which is thought to result from ROS-induced changes to the N-terminus of albumin [143], and the IMA/albumin ratio are elevated in sera of patients with stable RRMS compared to control subjects [144].

Elevated levels of homocysteine have been associated with vascular injury and atrophy (or neurodegeneration) of some CNS structures [145, 146], and homocysteine and cysteine plasma levels are elevated in MS [147-149]. Albumin can regulate thiol/disulfide exchange reactions in plasma, and albumin is the main protein that binds homocysteine in plasma [147, 150, 151]. It has been put forth that homocysteine might promote toxicity to the CNS [146]; if so, then albumin could influence this process. For instance, the concentration of homocysteine is much greater in plasma than CSF [146]; thus, albumin might act as a carrier delivering homocysteine to the CNS at times of BBB leakage in MS and potentially worsening pathology. On the other hand, the binding homocysteine to albumin, and the rapid clearance of albumin from the CNS, e.g., in active lesions (discussed above), could limit the ability of homocysteine to promote toxicity.

### The putative synthesis of albumin by microglia

Besides accessing the CNS following BBB leakage, albumin may be produced within the CNS. An in vitro and tissue study found that human microglia produce albumin and the expression increases upon microglial activation [152]. Albumin induction in the CNS was also observed following ischemia [153, 154]. Albumin produced by microglia was postulated to have a protective role in Alzheimer’s disease by blocking the polymerization of amyloid beta and facilitating its clearance [152], and albumin produced in the CNS could have additional protective properties equivalent to those discussed above. However, in follow up studies, albumin that has become glycated was shown to be produced by rat and/or human microglial cells that were treated with amyloid beta or ethanol, and glycated albumin was suggested to promote neurodegeneration [155, 156]. It is not known if glycated albumin is produced by microglial cells in MS. An elevation of glycated products has been observed in MS tissue [157, 158], but it is not clear if albumin was among the products, or if glycated products came from extravasation through a leaky BBB or were produced endogenously, e.g., in microglia. When albumin becomes glycated it diminishes its anti-oxidant properties [159–161], and in vitro studies found that exposure of rat retinal microglial cells to glycated albumin resulted in microglial production of proinflammatory cytokines, TNFα and IL-1β [162]. The expression of these cytokines is increased in MS [163] and may contribute to MS pathology including enhancing leakage of the BBB [136, 164]. Thus, glycated albumin might be in a position to worsen disease activity in MS, although it is unclear what the outcome would be if other molecules became glycated in place of albumin.

In contrast to glycated albumin, production of non-glycated albumin in the CNS is likely protective. However, it is still unclear whether albumin is produced by CNS cells in MS. In one study, albumin was detected within astrocytes, oligodendrocytes, axons, and macrophages in inactive plaques [7], which would support in situ synthesis, but additional studies are warranted, especially on active plaques, in order to fully establish which cells, if any, produce albumin in MS. Furthermore, even if albumin is synthesized in the CNS, it is unclear whether the amount produced will be sufficient to substantially influence albumin levels in the CSF or albumin levels in the CNS resulting from a breach in the BBB.

### Albumin as a therapy in models of CNS diseases

Albumin administered in high doses has been examined for therapeutic value in models of some CNS diseases. In models of ischemia (e.g., global, transient focal, or permanent focal) or traumatic brain injury, administration of albumin resulted in protection, e.g., reduced infarction volume [165, 166]. Most of the benefits are thought to be related to hemodynamics effects, such as expanding the volume of the blood, but other mechanisms (similar to those discussed above) could also have protective roles [165]. In addition, the binding of albumin to megalin, a receptor on astrocytes [167], stimulates cultured astrocytes to produce oleic acid, which might support neuronal differentiation during development [168, 169]. Albumin also binds oleic acid [170], and treatment with albumin or albumin-oleic acid promoted recovery [171, 172] and decreased microglial activation [172] following spinal cord injury in the rat.

Although it is possible that administration of exogenous albumin or albumin-oleic acid may confer some benefits for MS similar to the preclinical results described above for other conditions, high dose albumin therapy has been tested in clinical trials for ischemic stroke but was found to have no benefit, and it even increased mortality in subjects that were > 83 years old [173]. The increased mortality in elderly individuals was suggested to be due to increased myocardial stress [174]; of note, there is evidence to suggest that MS patients have a greater risk for myocardial infarction [175]. Thus, great caution should be taken before pursuing a similar strategy in MS even if pre-clinical studies provide encouraging results.

### Possible pathogenic roles of albumin during MS

Some studies obtained results suggesting that albumin has the potential to worsen disease activity in the CNS. For instance, injection of albumin into the neostriatum in one hemisphere resulted in a greater lesion volume compared to injection of saline into the other hemisphere in the rat [176] while infusion of serum or serum fraction(s) in CA1 sector or striatum led to neuronal loss or inflammatory lesions in these respective locations [177]. However, the outcomes observed in these studies could have been due to factors other than the injected albumin or serum components. For instance, the osmotic effects of albumin or serum proteins, and/or the large volume of injected material may account for the lesions since a slow injection of plasma proteins into the hippocampus did not cause neurodegeneration [178]. In another study, an intracerebroventricular injection of albumin didn’t result in neurodegeneration in rats, however, when it was combined with hippocampal administration of kainic acid, which is used to induce seizures, neurodegeneration of CA3 neurons was enhanced compared to kainic acid plus vehicle [179].

Seizure activity is associated with an opening of the BBB, and extravasation of albumin into the brain is thought to increase the excitability of neurons and induce proinflammatory events. For example, a model of status epilepticus in rats resulted in extravasation of albumin in the hippocampus, which was diffusely distributed at 2 h post status epilepticus and became concentrated in CA3 neurons at 24 h [179]. An intracerebroventricular injection of albumin resulted in high frequency, high amplitude spiking activity in the rat hippocampus lasting 1 h after injection, and induced IL-1β expression by hippocampal astrocytes at 2 h, which was further increased by 24 h [179]. Besides induction in astrocytes, albumin can induce microglia to express IL-1β [180]. In studies involving CSF from MS patients with active disease, together with in vitro mouse brain slice preparations and other related analyses, IL-1β was found to be associated with inducing excitatory postsynaptic currents and glutamate excitotoxic neuronal damage [181]. Albumin can also induce other inflammatory responses by astrocytes and microglia. For example, albumin can induce astrocytic expression of CX3CL1 [180], which is a chemokine involved in CNS recruitment of CD4+ T cells in RRMS [182], and albumin can induce microglial activation, i.e., increase intracellular levels of calcium and proliferation [183]. Albumin can also induce astroglial and microglial expression of nitric oxide metabolites [180].

Both the prevalence and incidence of seizure disorders is greater in MS patients than in the general population [184], but even without seizures, it is possible that leakage of albumin through a damaged BBB in MS could lead to some similar pathogenic events that occur in epilepsy. Epilepsy results in BBB leakage and extravasation of albumin into CNS structures, and the presence of albumin is thought to exacerbate disease by inducing inflammatory events (discussed above) and by altering potassium homeostasis [76]. In the CNS, extravasated or exogenously administered albumin results in its presence in the parenchyma and uptake by microglia, astrocytes and neurons [73–76] leading to astrocyte gliosis and neuronal loss [185]. Mechanistically, albumin is thought to enter astrocytes after interacting with TGF-β receptors and this uptake affects calcium concentrations in the cytoplasm [186] and results in the downregulation of Kir4.1 in astrocytes [73, 76, 179]. Extracellular potassium homeostasis becomes disrupted [73, 187] and neurons may become hyper-excitable to NMDA receptor activation [73, 76]. Also, albumin in neurons can increase the synthesis of glutamate [188] furthering this cycle. Since glutamate has been implicated in neurodegeneration in MS [189], it suggests that albumin could amplify pathology via this mechanism involving disruption of potassium homeostasis leading to greater sensitivity to glutamate.

Kir4.1 is localized to oligodendrocytes, perivascular astrocytes, astrocyte endfeet, and astrocyte processes associated with synapses [190, 191]. Interestingly, autoantibodies to Kir4.1 are at higher levels in MS than in controls, and are increased during a disease relapse compared to remission [192, 193], but their presence and/or role in MS have been questioned [194–196]. The expression of Kir4.1 is altered in MS lesions; in acute or chronic active demyelinating lesions Kir4.1 levels decreased while periplaque reactive astrocytes had increased levels [191]. Similar to its actions in epilepsy, it is possible that extravasated albumin can influence Kir4.1 expression in MS, and further dysregulation of Kir4.1 expression could facilitate the neurodegenerative process.

## Conclusions

In MS, CNS cells can become bathed by albumin in the context of ongoing inflammation following BBB disruption. Although many functions have been attributed to albumin, its role in MS has received little attention.

Damage to the BBB in MS is relatively common, and given the high concentration of albumin in plasma, it readily passes from the circulation into the CNS during BBB leakage. Although the albumin quotient is used as an indication of blood-CSF dysfunction, many factors can influence the CSF concentration of albumin indicating that this measurement is an imprecise indicator of BBB leakage or blood-CSF dysfunction. Once albumin becomes extravasated into the CNS, it can exert beneficial and/or harmful effects. Beneficial actions include albumin being a target for ROS and RNS, and in so doing limiting damage to other molecules. Albumin can also reduce the production of ROS and RNS by binding iron and heme. Despite these protective properties, albumin may promote pathology by acting to induce the production of proinflammatory cytokines, or disrupting potassium homeostasis making neurons potentially more vulnerable to glutamate excitotoxicity. Given the abundance of albumin together with frequent disruptions to the BBB, further studies are warranted to advance the understanding of the impact that albumin has on cellular functions and pathogenic processes in the context of MS. Some other pertinent areas of research include investigations on the roles of albumin variants, modified albumin (e.g., glycated albumin), and albumin levels on the disease course. Additionally, detailing the role of albumin in relation to the delivery or action of disease modifying therapies, and other drugs that are used to treat MS patients, is of interest.

In summary, given that albumin represents such a large percentage of the proteins that become extravasated during BBB leakage, albumin likely performs a multitude of roles that are largely dependent on the microenvironment albumin becomes exposed to during disease.

## Abbreviations

BBB: blood–brain barrier
CNS: central nervous system
EAE: experimental autoimmune encephalomyelitis
Gd: gadolinium
IMA: ischemia modified albumin
MS: multiple sclerosis
PPMS: primary progressive multiple sclerosis
RBCs: red blood cells
RNS: reactive nitrogen species
ROS: reactive oxygen species
RRMS: relapsing remitting multiple sclerosis
SPMS: secondary progressive multiple sclerosis
